# Major differences in follow-up practice of patients with colorectal cancer; results of a national survey in the Netherlands

**DOI:** 10.1186/s12885-019-6509-0

**Published:** 2020-01-06

**Authors:** S. M. Qaderi, N. A. T. Wijffels, A. J. A. Bremers, J. H. W. de Wilt

**Affiliations:** 1 Department of Surgical Oncology, Radboud university medical center, Geert Grooteplein Zuid 10, 6525 GA Nijmegen, The Netherlands; 2 Taskforce Coloproctology, Dutch Society of Surgery, Utrecht, The Netherlands

**Keywords:** Follow-up studies, Surveillance, Colorectal cancer, Survivorship, Survey

## Abstract

**Background:**

The precise content and frequency of follow-up of patients with colorectal cancer (CRC) is variable and guideline adherence is low. The aim of this study was to assess the view of colorectal surgeons on their local follow-up schedule and to clarify their opinions about risk-stratification and organ preserving therapies. Equally important, adherence to the Dutch national guidelines was determined.

**Methods:**

Colorectal surgeons were invited to complete a web-based survey about the importance and interval of clinical follow-up, CEA monitoring and the use of imaging modalities. Furthermore, the opinions regarding physical examination, risk-stratification, organ preserving strategies, and follow-up setting were assessed. Data were analyzed using quantitative and qualitative analysis methods.

**Results:**

A total of 106 colorectal surgeons from 52 general and 5 university hospitals filled in the survey, yielding a hospital response rate of 74% and a surgeon response rate of 42%. The follow-up of patients with CRC was mainly done by surgeons (71%). The majority of the respondents (68%) did not routinely perform physical examination during follow-up of rectal patients. Abdominal ultrasound was the predominant modality used for detection of liver metastases (77%). Chest X-ray was the main modality for detecting lung metastases (69%). During the first year of follow-up, adherence to the minimal guideline recommendations was high (99–100%). The results demonstrate that, within the framework of the guidelines, some respondents applied a more intensive follow-up and others a less intensive schedule. The majority of the respondents (77%) applied one single follow-up imaging schedule for all patients that underwent treatment with curative intent.

**Conclusions:**

Dutch colorectal surgeons’ adherence to minimal guideline recommendations was high, but within the guideline framework, opinions differed about the required intensity and content of clinical visits, the interval of CEA monitoring, and the importance and frequency of imaging techniques. This national survey demonstrates current follow-up practice throughout the Netherlands and highlights the follow-up differences of curatively treated patients with CRC.

## Background

Colorectal cancer (CRC) is the third most common cancer in both men and women [1]. Over the years, survival has improved significantly in patients with CRC resulting in survivors in follow-up [1, 2]. After treatment, patients are followed to detect and treat early disease recurrence or metastases. Moreover, the goal of surveillance is to consult patients regarding complications, adverse effects, and prognostic information [3]. In the Netherlands, follow-up of patients with CRC occurs within the framework of the national guidelines and usually consists of at least biannual clinical visits and laboratory and imaging tests (Additional file 1).

The goal, frequency and content of outpatient visits are a subject of debate in many cancer types and scheduled out-patient visits are generally of limited value [4–7]. Still, follow-up of patients with CRC is useful [8]. Follow-up modalities such as serum CEA are helpful to shorten the lead time to diagnosis of recurrent disease. However, the effect of earlier detection on overall survival is limited [5, 8]. Intensive follow-up schedules fail to produce significantly improved outcomes [9, 10]. Inevitably, intensive surveillance results in higher cost [11], radiation exposure and discomfort.

A recent review of European guidelines conducted by Bastiaenen et al. demonstrated that although multimodal follow-up after curative treatment of CRC is recommended in all countries, the precise content and intervals of these modalities are variable [12]. The Dutch national guidelines for CRC have similarities with the European Society for Medical Oncology (ESMO) and American Society of Clinical Oncology-American Society of Colon and Rectal Surgeons (ASCO-ASCRS) guidelines. Nevertheless, there are also some differences between above guidelines. For instance, Carcino-Embryogenic Antigen (CEA) monitoring is recommended 3-to-6 monthly in the first 3 years and 6-to-12 monthly hereafter in the Netherlands. Another difference between European and American guidelines is the use of ultrasound in Europe in contrast to routine Computed-Tomography (CT) of the abdomen as recommended in the American guidelines. Without consensus about the content of follow-up schedules and the existence of different guidelines, practice between surgeons and hospitals varies. A study by Grossmann et al. demonstrated that surgeon adherence to national guidelines was low in the Netherlands, and an American study shows low adherence to follow-up guidelines too [13, 14]. Similar variations have also been reported in follow-up of melanoma patients by surgeons and dermatologists [6]. The status of follow-up adherence in the Netherlands has not been studied since 2007, while clinical guidelines have changed. Hence, information about the current status of follow-up is needed.

The aim of this study was to assess the view of Dutch Colorectal Surgeons on their local follow-up schedule, clarify their opinions about risk-stratification and organ preserving therapies. Equally important, adherence to the Dutch national guidelines was determined.

## Methods

### Procedure and participants

All members of the Dutch Taskforce Coloproctology (WCP: Werkgroep Coloproctologie) were invited to complete a web-based survey in October and November 2018. Invitations were sent by email with the purpose of the survey, a request to participate anonymously, and the hyperlink to the actual questionnaire. Only colorectal surgeons treating patients with CRC were included. (*N* = 250). Two reminders were sent; one after 2 weeks, and a final reminder in which closing of the survey was announced and invitees were requested to either fill in the survey or finish their survey. The survey was locked after completion in order to prevent answer changes.

### Survey

A web-based survey was developed using Castor EDC (Castor Electronic Data Capture, Ciwit BV, Amsterdam, The Netherlands, 2018). The survey was conducted in Dutch and consisted of 36 multiple-choice questions, 2 open questions and another 9 open and 16 multiple-choice additional questions depending upon previous answers (Additional file 2: English translation). The questionnaire included questions regarding the following fields: local follow-up schedule, diagnostic modality, duration and intensity of follow-up and population followed. Furthermore, the opinions regarding physical examination, risk-stratification, and organ preserving strategies were assessed. In addition, opinions regarding ten carefully formulated statements were determined on a five-point Likert scale. At the end of the questionnaire, the opportunity was given to state comments or ask additional questions.

Curative CRC surgery was defined as surgical treatment of stage I-III disease according to the American Joint Committee on Cancer (AJCC) classification. Adherence to the national guidelines was defined as the percentage that adhered to the minimal recommendations. (i.e., biannual clinical visit, CEA monitoring and abdominal ultrasound) Least intensive clinical follow-up was defined as two clinic visits, two yearly CEA sampling and two-yearly use of abdominal US. Most intensive follow-up was defined as performing four clinical visits, four CEA measurements, and two-yearly US.

### Statistical analysis

In this qualitative study, descriptive statistics were used. Data were depicted as frequencies and percentages. Data from open questions, comments and views were analyzed in a qualitative fashion.Analyses were done using SPSS (version 23.0; IBM Corp, Armonk, NY).

## Results

### Respondents‘characteristics

A total of 106 colorectal surgeons from 52 general and 5 university hospitals in the Netherlands filled in the survey, yielding a hospital response rate of 74% and a surgeons response rate of 42%.

### Outpatient clinic visit and physical examination

The follow-up of patients with CRC was mainly done by surgeons (71%). Other involved clinicians were nurse practitioners (10%), gastroenterologists (3%), surgical residents (2%), or a combination of the above (14%). Colonoscopy surveillance was mainly performed by gastroenterologists (87%). More than half (55%) of the respondents did not follow patients with pT1N0 colorectal disease at the outpatient clinic. These patients were only followed according to the colonoscopy surveillance guideline but did not undergo regular visits, CEA, or imaging tests.

The majority (55%) reported that geriatric patients were followed regardless of their age, while others ended surveillance of patients older than 80 (8%), 85 (19%) or 90 years (11%).

Physical examination was not routinely performed by 68 and 67% of the respondents in patients with colon or rectal cancer, respectively. Components of physical examination are shown in Table 1.

**Table 1:** Performance of the different components of physical examination (PE) at outpatient visits of colon and rectal cancer patients

	Colon cancer patients % (*N* = 106)	Rectal cancer patients % (*N* = 106)
No PE	67	68
Abdominal PE and DRE together	4	22
Abdominal PE alone	23	7
DRE only	5	1
DVE	0	1
Other i.e. Inguinal (lymph node) examination	1	1

Numbers represent percentages

*DRE* digital rectal examination, *DVE* digital vaginal examination

Questionnaires are taken routinely in 24% of the cases during clinical visits, mainly European Organization for Research and Treatment of Cancer (EORTC)-Quality of Life forms (4%), distress thermometer and problem list (15%), Brief Pain Inventory or other related pain questionnaires (1%), patient satisfaction surveys (2%), adverse event/complication questionnaires such as the Low Anterior Resection Syndrome Score (13%), or other Patient Related Outcome Measure questionnaires (4%).

### Tumor marker blood measurement

CEA was routinely used as a tumor marker (99%) during follow-up. One respondent applied an intensive CEA measurement protocol, with blood sampling every 6 weeks during the first 2 years and every 3 months hereafter (Table 2).

**Table 2:**
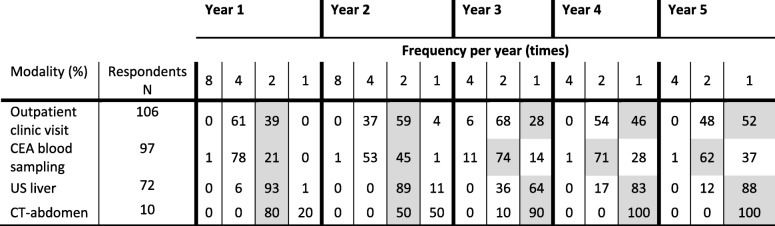
Follow-up schedule according to current practice (% of the participants) and the actual frequency recommended by the national guidelines (gray) among colorectal surgeons

Numbers represent percentage of respondents that performed the modality. The number of respondents is equal for all years

*CEA* Carcinoembryonic antigen, *US* Ultrasound, *CT* Computed-Tomography

### Imaging modalities

Abdominal ultrasound was the predominant modality used for detection of liver metastases during follow-up (77%), followed by CT-scan (11%) or a combination of both (10%) with the remaining 2% being “otherwise, not specified”. US is generally performed every 6 months in the first 2 years and yearly hereafter (Table 2).

If used, CT-abdomen was performed routinely during the first 4 years, usually with a 6-month interval (Table 2). The following reasons for using CT-abdomen instead of US were reported. First, US was technically not feasible (42%). Second, it was a matter of local protocol without providing further explanation (40%). Third, US has lower sensitivity compared to CT (11%). Fourth, being specified as “other” (7%). Adherence to minimal guideline recommendations (at least biannually) regarding clinical visit (100%), CEA sampling (100%), and liver US (99%) was high during the first year.

Surgeon reported free text answers reasoning the use of CT-abdomen, were as follows (*N* = 21):

- In rectal cancer follow-up, preferably after 1–2 years of follow-up (24%)
- In high-risk patients or young patients (19%)
- Intermittent CT-abdomen/US liver (14%)
- Follow-up of specific findings/lesions (9%)
- When CT performed for other reasons such as follow-up of atypical lung lesions (9%)
- Elevated CEA (5%)
- Adiposity/ hepatic steatosis (5%)
- Determined by multidisciplinary team (5%)
- CT at diagnosis for comparison purposes (5%)
- When indicated, “not otherwise specified” (5%)

The majority of the respondents (77%) applied one single follow imaging schedule for all patients that underwent treatment with curative intent. However, they did not use a uniform schedule; 26% had a different scheme for high-risk patients citing various definitions (Table 3).

**Table 3:** Definition of high-risk CRC patients according to surgeons (% of total responses). Total number of text citations was 41 from 24 different surgeons

Subject	Definition	Percentage (%)
Lymph node status	Harboring positive nodal stage (Nany)	31.7
Tumor characteristics	Harboring large size tumors (T4)	21.9
	Aggressive tumor n.o.s.	
Disease stage	Stage II (high-risk) – III disease	17.1
	LARC	
	M1 disease (curatively treated)	
Histology characteristics	Lymph vessel invasion	12.2
	Venous invasion	
	R1/2 resection	
Intra-operative characteristics	Tumor spill, perforated tumor, findings n.o.s.	7.3
Adjuvant therapy	Absence of CRT	4.9
Pre-operative diagnostics	Atypical lesions liver and/or lungs on CT	4.9

Responses were categorized by subject

*CT* Computed-Tomography, *T* Tumor size according to AJCC TNM classification, *LARC* Locally Advanced Rectal Carcinoma, *n.o.s* Not otherwise specified, *CRT* Chemoradiation therapy

In high-risk patients, there is a higher tendency to perform physical examination including DRE or colonoscopy, to use CT-thorax/abdomen instead of US and chest X-ray, and to perform pelvic MRI or PET-CT scanning. Also, several colorectal surgeons reported intensifying the diagnostic modality interval (CEA, CT thorax/abdomen) to every 3–4 months in the first 2 years of follow-up or at least perform a yearly CT-thorax/abdomen in high-risk patients.

### Diagnostic imaging for detecting distant metastases

The majority of the respondents (82%) made routine use of imaging to detect lung metastases in rectal cancer patients. Chest X-ray was the main diagnostic modality according to 69% of the respondents, while the remaining 31% used CT-thorax.

### Organ preserving therapy

Most respondents (59%) provided rectum preserving treatment in their own institution. Almost all of the rectal cancer patients (96%) that had been treated with neo-adjuvant radiotherapy (5x5Gy) or neo-adjuvant (long course) radiation and chemotherapy underwent a routine preoperative restaging pelvic MRI on a routine basis, while only 29% of the respondents use endoscopy to assess tumor response. Almost all surgeons (92%) provide follow-up according to the Wait and See protocols as alternative treatment for surgery in selective patients.

**Fig. 1:**
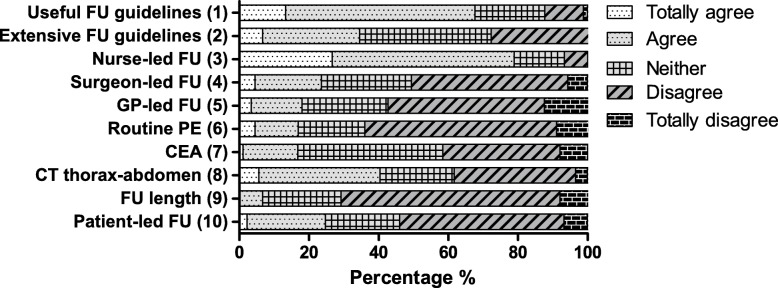
Agreement of participants regarding statements (%). 1: The current national CRC guidelines are clear and useful. 2: The current national CRC guidelines are too complicated and could be more concise. 3: Follow-up of patients with CRC can be done by nurse practitioners and/or case managers. 4: Surgeons should be the primary responsible clinicians for CRC follow-up. 5: General practitioners are well able to take over the CRC follow-up. 6: Physical examination should be performed routinely during follow-up of patients with CRC. 7: There is enough evidence that only CEA monitoring is cost-effective and useful in colorectal follow-up. 8: Patients with CRC should have a CT- thorax/abdomen at 12- and 24-months post-treatment to detect metastasis early. 9: Colorectal follow-up can be finished after 2 years because there is low risk of disease recurrence. 10: Patients with CRC are well able to coordinate their own follow-up and appointments

Surgeons opinions about current practice and developments. Surgeons expressed their opinion regarding the ten statements below (Fig. 1, *N* = 89).

1. The current national CRC guidelines are clear and useful
2. The current national CRC guidelines are too complicated and could be more concise
3. Follow-up of patients with CRC can be done by nurse practitioners and/or case managers
4. Surgeons should be the primary responsible clinicians for CRC follow-up
5. General practitioners are well able to take over the CRC follow-up
6. Physical examination should be performed routinely during follow-up of patients with CRC
7. There is enough evidence that only CEA monitoring is cost-effective and useful in colorectal follow-up
8. Patients with CRC should have a CT- thorax/abdomen at 12- and 24-months post-treatment to detect metastasis early
9. Colorectal follow-up can be finished after 2 years because there is low risk of disease recurrence
10. Patients with CRC are well able to coordinate their own follow-up and appointments

Cost differences between most intensive and least intensive clinical follow-up*.* (According to the Dutch Healthcare Authority [15]).

Based on the national average cost of an outpatient clinic visit (€195), CEA blood sampling (€8), abdominal US (€93), and chest X-ray (€44), the total cost difference between least and most intensive follow-up of patients with colon or rectal cancer in the first year of follow-up was €406 (Table 4) Personnel costs, overhead costs and parking/transport costs for patients are not included in this calculation.

**Table 4:** Cost differences between least and most intensive clinical follow-up of patients with colon or rectal cancer

Follow-up regimen	Estimated annual costs (€)	
	Colon cancer	Rectal cancer
Least intensive	592	680
Most intensive	998	1086
Cost difference	406	

Least intensive: Defined as two clinical visits, two-yearly CEA sampling and two-yearly use of abdominal US. Most intensive: Defined as four clinical visits, four CEA measurements, and two-yearly US

### Opinions about the current survey and national CRC guidelines

In total, 33 respondents expressed their opinion about the subject of this study, generally supporting its goals. Around one third (30%) of the respondents wrote that tailored follow-up schedules accounting for disease stage, patient age and clinical condition are required. According to them, the national guidelines are designed as a single size to fit all patients. Three respondents noted that follow-up is not always useful since there is no survival benefit of intensive follow-up schedules. Two respondents felt that the current guidelines are outdated and too much determined by radiation oncologists. Respondents called for the development of personalized follow-up strategies. Around 39% of them requested more research into setting of follow-up (GP, hospital), the value of separate elements of the follow-up schedule, and the required duration of surveillance.

## Discussion

The findings of this survey among colorectal surgeons in the Netherlands demonstrate differences and variety in current follow-up of patients with CRC that have undergone curative surgery. Adherence to minimal guideline recommendations was high. With a hospital response rate of 74%, including university centers, the survey was representative for daily practice throughout our country.

The current study demonstrates that in the Netherlands, follow-up is primarily performed by surgeons. Although there is some evidence that follow-up improves survival [8], there is no consensus about who should perform it. CRC is a common disease and patient follow-up can overwhelm clinic services, compromising the ability to see new referrals or reassess patients. Nurse-led follow-up can be a feasible alternative to consultant follow-up [16, 17]. The vast majority of this survey’s respondents agreed that follow-up can be done by specialized nurses, although in daily practice only 10% of the follow-up was done by nurses. Studies from Sweden and New Zealand demonstrated that follow-up can be performed by specialized nurses with equal high patient satisfaction and without compromising survival outcome [16, 17].

From a patient perspective, there is also willingness regarding non-physician follow-up such as GP-led or nurse-led follow-up [17–19]. Different views exist regarding a central the role of GPs in CRC follow-up. Some studies report positive views [18], while others demonstrate unfavorable views for a central role of GPs in follow-up [20, 21]. The majority of our respondents disagreed with GP-led follow-up. We noted that two-third of our respondents did not perform routine physical examination in stage I-III patients with colon or rectal cancer during follow-up. In contrast to our expectations, only 23% performed digital rectal examination in rectal cancer patients. The Dutch guidelines do not mention the value of DRE during follow-up. However, it is recommended in other European countries and local protocols [12].

Despite the little existing agreement between different European guidelines, most recommend clinical visits, CEA monitoring and liver imaging as part of follow-up [12]. This survey’s results showed a heterogeneous practice of national guidelines, but CEA was used as a diagnostic tool by all respondents either every 3 months or 6 months in the first 2 years. The CEA watch trial conducted in the Netherlands demonstrated that intensive CEA sampling detects recurrent disease earlier than standard protocols [5]. However, this regimen did not impact overall survival. In our survey, only one respondent performed intensive CEA monitoring. Ultrasound of the abdomen, according to our results, is the standard modality for detecting hepatic metastasis in the Netherlands. With the introduction of CT-techniques and the functional aspect of US (time consuming, inconclusive in the growing obese population), US may become increasingly limited. Currently, CT-scanning is the standard modality for stage I-III disease in the ASCO, ASCRS, and UK guidelines but can be substituted by liver US in ESMO guidelines [8].

The Dutch guidelines recommend using the high-risk criteria of the ASCO guidelines for identifying high-risk stage II disease patients. Our results revealed heterogeneity in defining high-risk patients. Some participants in our survey based high-risk status on tumor size, biology, and histopathological characteristics, while others based it on intra-operative findings and adjuvant treatment. This variation underlines the need of guidelines and clear definitions regarding low and high-risk status.

Respondents mentioned that tailoring means stratifying by disease stage, age, clinical condition, and patient preferences and capacities. In geriatric patients with high co-morbidity burden, surgeons considered ending follow-up since the high mortality and morbidity related to secondary or salvage surgery for recurrent disease outweighs the advantages of routine follow-up. Recently, several cost effectiveness studies about follow-up schedules have been performed. In a randomized clinical trial of Mant et al. high intensity follow-up after curative colorectal surgery was costly and did not have any impact on survival or QALYs [11]. For that reason, we estimated the potential costs of minimal and maximal adherence to national guidelines as reported in this survey.

We calculated an estimated difference of €406 per patient, per year between minimal and maximal follow-up. These costs represent only the costs for the laboratory and imaging costs, without considering the potential cost minimization of implementing nurse-led instead of surgeon-led follow-up. Since many studies have shown that there is no survival benefit of costly, intensive follow-up [3, 9, 11] future research is needed to investigate cost effective, personalized follow-up schedules [22].

The Dutch guidelines allow, to a certain extent, for different interpretations and therefore variation in practice. Also, surgeons can deviate from the guidelines as long as they demonstrate due care and consideration. Knowing that our survey respondents adhered strongly to the guidelines, it is interesting to note that there is variation in content and frequency of follow-up modalities. Several factors might have contributed to this variety. First, the presence of broad and less detailed follow-up guidelines. Second, the existence of intra- and inter-surgeon variation in the interpretation of, and adherence to guidelines. Third, variation in patient selection.

A strong aspect of this survey is the fact that it demonstrates current follow-up practice throughout the Netherlands and highlights the differences regarding duration, content and intensity of follow-up. Nevertheless, there are some limitations. First, surveys harbor potential reporting bias since self-formulated questions and statements can be interpreted differently by respondents or yield socially desirable answers. We cautiously formulated the questions and tried to formulate non-ambiguous and non-directive questions. Second, the surgeon response rate was relatively low, but hospital response rate was high. The survey yielded response from hospital representatives from various regions of the country. Moreover, comparable surgeon response rates were reported in other studies [21, 23–26].

## Conclusions

Adherence to national follow-up guidelines was high although guidelines are interpreted differently by colorectal surgeons working in the Netherlands. Dutch colorectal surgeons practiced and thought different about the intensity and content of clinical visits, the interval of CEA monitoring, the importance and intensity of imaging techniques such as ultrasound and computed-tomography. Also, heterogeneity exists in defining high-risk status of patients with CRC.

In summary, this national survey demonstrates current follow-up practice throughout the Netherlands and highlights the follow-up differences of curatively treated patients with CRC.

## Supplementary information

**Additional file 1.** Short outline of the Dutch guidelines.

**Additional file 2.** English translated copy of the actual Online survey (held in Dutch).

## Data Availability

The datasets used and/or analyzed during the current study are available from the corresponding author on reasonable request.

## Abbreviations

CEA: Carcino-Emryogenic Antigen
CRC: Colorectal cancer
CT: Computed-Tomography
WCP: Dutch Taskforce Coloproctology (Dutch: Werkgroep Coloproctologie)
