# Supplementary material for: Major differences in follow-up practice of patients with colorectal cancer; results of a national survey in the Netherlands
Source: BMC Cancer. 2020 Jan 6;20:22. doi: 10.1186/s12885-019-6509-0 (PMC6945647; doi:10.1186/s12885-019-6509-0)
Supplement: Supplementary file 1 — Additional file 1. Short outline of the Dutch guidelines. [file 12885_2019_6509_MOESM1_ESM.pdf]

Supplement 1. Short outline of the Dutch guidelines

Last update in 2014; the Dutch CRC guidelines recommends a half-yearly outpatient clinic visit for 2-3 years postoperative and yearly hereafter up to 5 years. CEA measuring is recommended 3-to-6 monthly up to 3 years and half-yearly hereafter. Furthermore, abdominal ultrasound, CT-abdomen and chest X-ray (rectal cancer only) are recommended every half-year up to three years and yearly hereafter. The Dutch Colonoscopy Surveillance guideline recommends colonoscopy at 1, 4- and 9-years post-operative. In case of incomplete pre-operative colonoscopy; a complete colonoscopy should be done within 3 months postoperatively.

Weblink reference (in Dutch):
https://www.oncoline.nl/colorectaalcarcinoom
