# Supplementary material for: Major differences in follow-up practice of patients with colorectal cancer; results of a national survey in the Netherlands
Source: BMC Cancer. 2020 Jan 6;20:22. doi: 10.1186/s12885-019-6509-0 (PMC6945647; doi:10.1186/s12885-019-6509-0)
Supplement: Supplementary file 2 — Additional file 2. English translated copy of the actual Online survey (held in Dutch). [file 12885_2019_6509_MOESM2_ESM.pdf]

Supplement 2. English translated copy of the actual Online survey (held in Dutch).

Survey 'Local follow-up practice'

Local follow-up practice - Hospital

Number	Question	Answers
1.1	In which hospital do you work?	

Local follow-up practice – General

Number	Question	Answers
2.1	Who is primarily responsible for follow-up of patients with non-metastasized disease?	

- ○ Surgeon
- ○ Gastroenterologist
- ○ Resident
- ○ Nurse practitioner
- ○ General practitioner
- ○ Combination of above
- ○ Otherwise

2.2	If ' Who is primarily responsible for follow-up of patients with non-metastasized disease?' is equal to 'Otherwise' answer this question:<br>Explain.	
2.3	If ' Who is primarily responsible for follow-up of patients with non-metastasized disease?' is equal to 'Combination of above' answer this question:<br>Which combination?	◯ Surgeon and resident<br>◯ Surgeon and nurse practitioner<br>◯ Gastroenterologist and resident<br>◯ Gastroenterologist and nurse practitioner<br>◯ General practitioner and medical specialist<br>◯ Other combination
	2.3.1 If ' Which combination?' is equal to 'Other combination' answer this question:<br>Explain	
2.4	What is the interval (months) of outpatient clinic visits during the first year?	◯ 3<br>◯ 6<br>◯ 12
2.5	What is the interval (months) of outpatient clinic visits during the second year?	◯ 3<br>◯ 6<br>◯ 12
2.6	What is the interval (months) of outpatient clinic visits during the third year?	◯ 3<br>◯ 6<br>◯ 12
2.7	What is the interval (months) of outpatient clinic visits during the fourth year?	◯ 3<br>◯ 6<br>◯ 12
2.8	What is the interval (months) of outpatient clinic visits during the fifth year?	◯ 3<br>◯ 6<br>◯ 12
2.9	Do you perform standard physical examination during follow-up of patient with non-metastasized disease?	◯ No (only if indicated)<br>◯ Yes

2.1	If ' Do you perform standard physical examination during follow-up of patient with non-metastasized disease?' is equal to 'Yes' answer this question:<br><br>Specify what type of physical examination is performed in patents with rectal cancer.	☐ Abdominal examination<br>☐ Rectal examination<br>☐ Vaginal examination (in women)<br>☐ Otherwise
2.1.1.1	If ' Specify what type of physical examination is performed in patents with rectal cancer.' is equal to 'Otherwise' answer this question:<br>Explain.	
2.10	If ' Do you perform standard physical examination during follow-up of patient with non-metastasized disease??' is equal to 'Yes' answer this question:<br>Specify what type of physical examination is performed in patents with colon cancer.	☐ Abdominal examination<br>☐ Rectal examination<br>☐ Vaginal examination (in women)<br>☐ Otherwise
2.1.1.2	If ' Specify what type of physical examination is performed in patents with colon cancer.' is equal to 'Otherwise' answer this question:<br>Explain	
2.2	Do you take routine questionnaires during the outpatient visit?	◯ No<br>◯ Yes
2.1	If ' Do you take routine questionnaires during the outpatient visit?' is equal to 'Yes' answer this question:<br>Which questionnaires?	☐ Quality of life (EORTC-QoL or others)<br>☐ Last meter/symptom burden thermometer<br>☐ Pain questionnaires (BPI)<br>☐ Fatigue (MFI)<br>☐ Patient satisfaction<br>☐ Symptom screening (LARS score, Bristol stool score, stoma lists)<br>☐ Other
2.2	Do you follow patients with T1N0 colorectal cancer in the outpatient clinic? (except colonoscopy)	◯ No<br>◯ Yes
2.3	Do you stop follow-up of patients above a certain age?	◯ No<br>◯ Yes

2.3.1	If ' Do you stop follow-up of patients above a certain age?' is equal to 'Yes' answer this question: If yes, specify from what age you do not follow-up patients	◯ 75<br>◯ 80<br>◯ 85<br>◯ 90

Local follow-up practice - CEA monitoring for stage I-III patients

Number	Question	Answers
3.1	Do you use CEA as a standard modality during follow-up?	◯ No<br>◯ Yes
3.2	What is the interval (months) of CEA monitoring during the first year?	◯ 1,5<br>◯ 3<br>◯ 6<br>◯ 12
3.1	What is the interval (months) of CEA monitoring during the second year?	◯ 1,5<br>◯ 3<br>◯ 6<br>◯ 12
3.2	What is the interval (months) of CEA monitoring during the third year?	◯ 3<br>◯ 6<br>◯ 12
3.3	What is the interval (months) of CEA monitoring during the fourth year?	◯ 3<br>◯ 6<br>◯ 12
3.4	What is the interval (months) of CEA monitoring during the fifth year?	◯ 3<br>◯ 6<br>◯ 12

Local follow-up practice – Imaging for stage

I-III patients

Number	Question	Answers
4.1	The imaging follow-up schedule is the same for all patients with stage I-III disease.	◯ No<br>◯ Yes
4.2	For high-risk patients there another follow-up schedules is applied.	◯ No<br>◯ Yes
4.2.1	If ' For high-risk patients there another follow-up schedules is applied.' is equal to 'Yes' answer this question:<br>Define your definition of high-risk.	
4.2.2	If ' For high-risk patients there another follow-up schedules is applied.' is equal to 'Yes' answer this question:<br>If another schedule, specify how this does like.	
4.3	What is the standard modality used for the imaging of the liver/abdomen?	◯ Echo (ultrasound/sonography)<br>◯ Computed-Tomography (CT)<br>◯ Combination of echo and CT<br>◯ Other
4.1	If ' What is the standard modality used for the imaging of the liver/abdomen?' is equal to 'Other' answer this question:<br>Explain:	
4.4	If ' What is the standard modality used for the imaging of the liver/abdomen?' is equal to ' Combination of echo and CT' answer this question:<br>What is the interval and intensity of this combination?	
4.1.1	If ' What modality is used standardly for the imaging of the liver/abdomen?' is equal to 'Echo' answer this question:<br>If echo-liver: What is the interval (months) during the first year?	◯ 3<br>◯ 6<br>◯ 12
4.1.2	If 'What modality is used standardly for the imaging of the liver/abdomen?' is equal to ' Echo' answer this question:<br>If echo-liver: What is the interval (months) during the second year?	◯ 3<br>◯ 6<br>◯ 12

4.1.1	If 'What modality is used standardly for the imaging of the liver/abdomen?' is equal to<br><br>' Echo' answer this question:<br>If echo-liver: What is the interval (months)<br><br>during the third year?	◯ 3<br>◯ 6<br>◯ 12
4.3.5	If 'What modality is used standardly for the imaging of the liver/abdomen?' is equal to<br>'Echo' answer this question:<br><br>If echo-liver: What is the interval (months) during the fourth year?	◯ 6<br>◯ 12
4.3.5	If 'What modality is used standardly for the imaging of the liver/abdomen?' is equal to<br>'Echo' answer this question:<br><br>If echo-liver: What is the interval (months) during the fifth year?	◯ 6<br>◯ 12
4.1.1	If ' What modality is used standardly for the imaging of the liver/abdomen?' is equal to<br>"Computed-Tomography (CT ' answer this question:<br>If CT: What is the interval (months)<br><br>during the first year?	◯ 3<br>◯ 6<br>◯ 12
4.1.2	If 'What modality is used standardly for the imaging of the liver/abdomen?' is equal to<br><br>'Computed-Tomography (CT)' answer this question:<br>If CT: What is the interval (months)<br><br>during the second year?	◯ 3<br>◯ 6<br>◯ 12
4.1.1	If ' What modality is used standardly for the imaging of the liver/abdomen?' is equal to<br>'Computed-Tomography (CT) ' answer this question:<br>If CT: What is the interval (months)<br><br>during the third year?	◯ 3<br>◯ 6<br>◯ 12
4.3.6	If 'What modality is used standardly for the imaging of the liver/abdomen?' is equal to<br>'Computed-Tomography (CT)' answer this question:<br><br>If CT: What is the interval (months) during the fourth year?	◯ 6<br>◯ 12
4.3.6	If 'What modality is used standardly for the imaging of the liver/abdomen?' is equal to<br>'Computed-Tomography (CT)' answer this question:<br><br>If CT: What is the interval (months) during the fifth year?	◯ 6<br>◯ 12

4.4	If CT-abdomen: Specify why CT.<br>○ Ultrasound technically not possible<br>○ No or insufficient experience with abdominal ultrasound<br>○ Ultrasound as lower sensitivity<br>○ According to local protocol without further explanation<br>○ Other	
4.5	If ‘CT-abdomen: Specify why CT?' is equal to 'other' answer this question:<br>Explain.	
4.6	Do you perform imaging to detect lung metastasis in patients with rectal cancer?	○ No<br>○ Yes
4.4	If 'Do you perform imaging to detect lung metastasis in patients with rectal cancer?' is equal to 'Ja' answer this question:<br>What is the standard modality for detecting long metastasis in patients with rectal cancer?	○ Chest X-ray<br>○ CT-thorax

Local follow-up practice - Colonoscopy

Number	Question	Answers
5.1	Which specialism does the endoscopic follow-up? (colonoscopy)	○ Surgery<br>○ Gastroenenterology

Local follow-up practice – Rectum sparing therapy

Number	Question	Answers
6.1	Do you perform standard restaging MRI after neoadjuvant therapy (radiation therapy (5x5Gy) and or chemoradiation?	○ No<br>○ Yes

6.2	Do you perform standard restaging endoscopy after neoadjuvant therapy (radiation therapy (5x5Gy) and or chemoradiation?	◯ No<br>◯ Yes
6.3	Do you provide "Wait and See" therapy for patients with early rectal cancer, as an alternative for standard TME surgery?	◯ No<br>◯ Yes
6.4	Do you refer patients eligible for organ preserving therapy to a tertiary center?	◯ No<br>◯ Yes

Local follow-up practice - Statements

Number	Question	Answers
7.1	The current national CRC guidelines are clear and useful.	◯ Totally agree<br>◯ Agree<br>◯ Neither<br>◯ Disagree<br>◯ Totally disagree
7.2	The current national CRC guidelines are too complicated and could be more concise.	◯ Totally agree<br>◯ Agree<br>◯ Neither<br>◯ Disagree<br>◯ Totally disagree
7.3	Follow-up of patients with CRC can be done by nurse practitioners and/or case managers.	◯ Totally agree<br>◯ Agree<br>◯ Neither<br>◯ Disagree<br>◯ Totally disagree
7.4	Surgeons should be the primary responsible clinicians for CRC follow-up.	◯ Totally agree<br>◯ Agree<br>◯ Neither<br>◯ Disagree<br>◯ Totally disagree

7.5	General practitioners are well able to take over the CRC follow-up.	◯ Totally agree<br>◯ Agree<br>◯ Neither<br>◯ Disagree<br>◯ Totally disagree
7.6	Physical examination should be performed routinely during follow-up of patients with CRC.	◯ Totally agree<br>◯ Agree<br>◯ Neither<br>◯ Disagree<br>◯ Totally disagree
7.7	There is enough evidence that only CEA monitoring is cost-effective and useful in colorectal follow-up.	◯ Totally agree<br>◯ Agree<br>◯ Neither<br>◯ Disagree<br>◯ Totally disagree
7.8	Patients with CRC should have a CT-thorax/abdomen at 12- and 24-months post-treatment to detect metastasis early.	◯ Totally agree<br>◯ Agree<br>◯ Neither<br>◯ Disagree<br>◯ Totally disagree
7.9	Colorectal follow-up can be finished after 2 years because there is low risk of disease recurrence.	◯ Totally agree<br>◯ Agree<br>◯ Neither<br>◯ Disagree<br>◯ Totally disagree
7.10	Patients with CRC are well able to coordinate their own follow-up and appointments.	◯ Totally agree<br>◯ Agree<br>◯ Neither<br>◯ Disagree<br>◯ Totally disagree

Local follow-up practice - Other

Number	Question	Answers

8.1	Do you have comments or feedback regarding this survey?	
